# Efficient Management of High-Frequency Sensor Data Streams Using a Read-Optimized Learned Index

**DOI:** 10.3390/s26041217

**Published:** 2026-02-13

**Authors:** Hu Luo, Jiabao Wen, Desheng Chen, Zhengjian Li, Meng Xi, Jingyi He, Shuai Xiao, Jiachen Yang

**Affiliations:** School of Electrical Automation and Information Engineering, Tianjin University, Tianjin 300072, China

**Keywords:** sensor data streams, IoT, learned index, spatial indexing, throughput

## Abstract

The rapid growth of sensor data in IoT and Digital Twins necessitates high-performance spatial indexing. Traditional indexes like Rtrees suffer from high storage overhead, while state-of-the-art learned indexes like GLIN encounter a “Refinement Bottleneck” due to coarse-grained Minimum Bounding Rectangle (MBR) filtering. Furthermore, existing solutions often trade update throughput for query accuracy, failing in dynamic IoT workloads with concurrent reads and writes. We propose DyGLIN (Dynamic Generate Learning-Based Index), a dynamic, read-optimized learned spatial index tailored for high-frequency sensor streams. DyGLIN introduces a decoupled leaf architecture separating query processing from data maintenance. To accelerate queries, we implement a hierarchical filtering pipeline using hierarchical MBRs (HMBR) and Cuckoo Filters to aggressively prune false positives. For maintenance, a Delta Buffer mechanism amortizes update costs, while logical deletion ensures high throughput. Experiments on real-world datasets show that DyGLIN reduces query latency by 26.4% [95% CI: 20.1%, 38.6%] compared to GLIN. It achieves 30.0% [95% CI: 21.4%, 35.9%] higher insertion throughput and superior deletion performance, with only an 18.5% [95% CI: 16.8%, 19.8%] increase in memory overhead.

## 1. Introduction

In the era of the Internet of Things (IoT) and Digital Twins, the ingestion of high-frequency sensor data streams has reached an unprecedented scale [1,2,3]. From the continuous stream of GPS trajectories [4,5] generated by ride-hailing platforms like Uber and DiDi to the complex polygon data for managing urban planning and environmental monitoring, spatial data management serves as the backbone of modern sensor-driven infrastructure [6,7]. This challenge is particularly pronounced in edge-centric environments where data must be processed in real time under dynamic workload conditions [8]. Unlike traditional relational data, sensor-captured spatial information is characterized by multi-dimensionality and complex geometric shapes (e.g., linestrings, polygons), which pose significant challenges for efficient real-time storage and retrieval.

For decades, spatial indexing in sensor systems has been dominated by tree-based structures such as Rtrees [9], Quad-Trees [10], and their variants (e.g., R*-Tree). These structures organize spatial objects into a hierarchy of Minimum Bounding Rectangles (MBRs) to facilitate range queries and k-nearest neighbor (kNN) searches. While robust, traditional indexes face inherent limitations in the context of “Big Spatial Data.” They typically rely on heuristic splitting algorithms that do not adapt to the underlying data distribution, leading to deep tree structures, excessive pointer chasing, and significant storage overhead [11]. As sensor data volume grows, these inefficiencies result in high I/O latency, creating a performance bottleneck for real-time sensor analytics.

To overcome the limitations of traditional structures, the database community has recently turned to learned indexes. Pioneered by Kraska et al. [12], this paradigm treats indexing as a machine learning problem. By training a model to learn the Cumulative Distribution Function (CDF) of the keys, learned indexes can predict record positions, significantly reducing storage and lookup time. Recent surveys highlight the rapid evolution of this field [13]. Significant progress has been made in extending these concepts to multi-dimensional data. For instance, Flood [14] and Tsunami [15] optimize data layout and grid structures for multi-dimensional query workloads, resulting in superior performance.

However, extending these advances to spatial data with complex geometries remains non-trivial. Early spatial learned indexes, such as the ZM-Index [16], LISA [17], and the recent RSMI [18] and WaZI [19], focus primarily on point data. They typically linearize spatial points using Space-Filling Curves (SFCs) or rank-space transformations. While effective for points, these approaches struggle with spatially extended objects like roads and lakes. The state-of-the-art solution, GLIN [20], addresses this by indexing Z-address intervals of MBRs. Despite its success, GLIN suffers from a critical “Refinement Bottleneck.” Its reliance on coarse-grained MBR approximation generates excessive “false positives”, which are objects that fall within the search range but do not spatially intersect the query window. Our analysis shows that in selective queries, the subsequent geometric refinement phase consumes over 70% of the total query time, which is unacceptable for latency-sensitive sensor feedback loops.

Furthermore, supporting efficient dynamic updates for continuous sensor streams remains a formidable challenge. While recent works like LIPP [21], FlexFlood [22], and USLI-DR [23] have begun to explore updatability in multi-dimensional spaces, applying these dynamic capabilities to complex geometries without compromising filtering precision remains an open problem. Existing solutions often trade ingestion throughput for query accuracy, making them unsuitable for hybrid IoT workloads where sensor data is continuously ingested while being queried.

To address these challenges, we propose DyGLIN, a dynamic and read-optimized learned spatial index specifically designed for sensor data management. DyGLIN adopts a “filter-heavy, write-optimized” philosophy and introduces a decoupled architecture at the leaf level. Our contributions are as follows:

Decoupled Architecture for Sensor Streams: We propose a novel leaf node structure integrating a hierarchical MBR (HMBR) and a Cuckoo Filter. This pipeline provides tighter spatial bounds, reducing the candidate set for expensive refinement by up to 71.8% [95% CI: 69.2%, 74.4%] and effectively solving the refinement bottleneck.

Buffered Maintenance and Logical Deletion: To handle high-frequency sensor updates, we introduce a Delta Buffer mechanism. Incoming writes are logged into a lightweight buffer to amortize the high structural update costs of HMBR. Additionally, we employ a logical deletion strategy using Cuckoo Filters, enabling O(1) deletion handling without immediate structural reorganization.

Comprehensive Evaluation: We evaluate DyGLIN against robust baselines on diverse real-world datasets. The results show that DyGLIN reduces query latency by 26.4% [95% CI: 20.1%, 38.6%] compared to GLIN by eliminating the refinement bottleneck. In terms of maintenance, DyGLIN achieves 30.0% [95% CI: 21.4%, 35.9%] higher insertion throughput than GLIN and superior deletion performance compared to all baselines, with only a modest 18.5% [95% CI: 16.8%, 19.8%] increase in memory overhead, proving its efficacy for high-performance hybrid workloads.

## 2. Related Work

Spatial indexing has been a cornerstone of database research for decades. The most prevalent structures are tree-based hierarchies, typified by the Rtree [9] and its variants (e.g., R*-Tree [24], R+-Tree [25]). Rtrees group spatial objects using MBRs and organize them into a balanced tree. While effective for general range queries, Rtrees suffer from performance degradation on massive datasets due to high MBR overlap and deep tree traversals [11]. Space-partitioning methods like Quad-Trees [10] and Grid Files recursively partition space but often struggle with data skew and high dimensionality. Recent work in edge computing has emphasized the importance of optimizing data flow pipelines for dynamic sensor environments [8]. However, these frameworks primarily focus on task scheduling and resource allocation at the system level, without addressing the fundamental challenge of efficiently indexing complex spatial geometries that arise in sensor-captured data.

The concept of learned indexes [12] proposes replacing internal tree navigation with machine learning models. Early works like the Recursive Model Index (RMI) demonstrated significant lookup speedups for 1D keys. Subsequent works have focused on supporting dynamic operations. ALEX [26] introduces gapped arrays to handle insertions, while the PGM index [27] provides worst-case bounds. More recently, RUSLI [28] uses spline-based models to support real-time updates, and LIPP [21] optimizes index structure for precise position prediction, further pushing the boundaries of updatable learned indexes.

Extending learned indexes to multi-dimensional data has attracted significant attention, as summarized in recent surveys [13]. The ZM-Index [16] and LISA [17] combine Space-Filling Curves (SFCs) with learned models to linearize spatial points. RSMI [18] introduces rank space-based ordering to learn spatial distributions directly without SFCs. ML-Index [29] utilizes iDistance to project points for learning. However, a common limitation of these approaches is their focus on point data. They cannot naturally handle complex geometries (e.g., polygons, linestrings) without reducing them to centroids, which leads to correctness issues in spatial range queries. WaZI [19] further optimizes the Z-index by making it workload-aware. However, these methods treat spatial objects as dimensionless points and cannot natively handle spatial extent.

Regarding multidimensional layout optimization, Flood [14] optimizes the index structure and data storage layout in conjunction with multidimensional range scans. Tsunami [15] improves upon Flood by handling correlated data and skewed query workloads. While they are highly efficient for tabular data, they lack support for geometric predicates (e.g., intersection, containment) required for complex spatial objects.

For non-point objects such as complex geometries, GLIN [20] indexes the Z-address range of the MBR. LSIR [30] attempts to improve efficiency by reusing the model, but it focuses primarily on static construction. As mentioned earlier, GLIN suffers from refinement bottlenecks due to the approximation of the MBR.

Most early works assume static data. Recent attempts like FlexFlood [22] and USLI-DR [23] have begun to explore dynamic maintenance for multi-dimensional learned indexes. However, applying these dynamic capabilities to complex geometries while maintaining high filtering precision remains an open problem. DyGLIN bridges this gap by integrating a decoupled architecture with Cuckoo Filters [31] and a buffered write mechanism, inspired by Write-Optimized Data Structures (WODS) like LSM-trees [32] and Buffer Trees [33].

## 3. Preliminaries and Problem Definition

### 3.1. Overview of Real-Time Spatial Queries for Sensors

We begin by formally defining the sensor-captured spatial dataset and the corresponding query operations:

- Spatial Dataset and Objects: Let $D=G_{1},G_{2},\ldots ,G_{N}$ be a dataset containing *N* complex spatial observations captured by sensors (e.g., environmental boundaries, vehicle trajectories, or obstacle zones). Each spatial observation $G_{i}$ is encapsulated by a Minimum Bounding Rectangle (MBR), denoted as $M_{i}$. While MBRs are efficient for coarse-grained indexing, they introduce approximation errors, especially for non-rectangular (e.g., diagonal or L-shaped) geometries.
- Spatial Range Query (SRQ): Given a query window *Q* representing a specific monitoring area or a safety perimeter, the goal of an SRQ is to retrieve the set *R* of all spatial objects that intersect with *Q* to enable immediate decision-making:

(1) $$R=\left\{G_{i}\in D|\mathrm{Intersection}\left(G_{i},Q\right)=\mathrm{True}\right\}$$

To facilitate efficient two-stage query processing, a Spatial Range Query *Q* is defined by a geometric region *r* (which can be an arbitrary polygon such as a monitoring area or safety perimeter). Query processing utilizes two representations of *Q*:

- *Q*.Geometry: The exact geometric shape *r* used for precise intersection tests in the refinement phase.
- *Q*.MBR: The Minimum Bounding Rectangle of *r*, denoted as MBR(r), used for index traversal and pruning in the filtering phase.

Formally, the result set *R* consists of all spatial objects o∈D such that their geometry intersects with *Q*.Geometry:

(2) R={o∈D∣Intersect(o.Geometry,Q.Geometry)}

The indexing phase, however, retrieves a superset candidate set *C* using the MBR approximation:

(3) C={o∈D∣Intersect(o.MBR,Q.MBR)}

where R⊆C. This classical filter-and-refine strategy ensures that *R* can be efficiently computed by first filtering to a small candidate set *C* using MBR-based indexing and then refining *C* through exact geometric verification.

### 3.2. The GLIN Data Retrieval Response Latency Model

The total response latency $T_{Query}$ for a spatial query is a critical metric. This latency can be decomposed into two main stages: the fast signal localization time $T_{Probe}$ and the precise verification time $T_{Refine}$:

(4) $$T_{Query}=T_{Probe}+T_{Refine}$$

- Probe Stage: The learned model is used to predict the index location (a range on the Z-order curve) of the MBR $M_{i}$. The time $T_{Probe}$ for this stage is typically very low, benefiting from the model’s predictive power and the efficiency of the underlying B-Tree or ALEX structure.
- Refine Stage: The located leaf node contains a candidate set *C*. The refine stage must perform expensive geometric intersection tests on each object in *C* to filter out false positives (FPs) and identify true positives (TPs).

### 3.3. Formalizing the Refinement Bottleneck

The refinement bottleneck is a critical latency barrier in learned-index-based sensor systems. It stems from the reliance on coarse-grained MBR approximations for initial filtering during the signal localization stage, which fails to provide sufficient precision for complex sensor-captured geometries.

In the context of sensor data management, the candidate set *C* is defined as the set of sensor observations for which their MBRs intersect with the monitoring window *Q*:

(5) $$C=\left\{G_{i}\in D|\mathrm{Intersection}\left(M_{i},Q\right)=\mathrm{True}\right\}$$

The precise verification latency $T_{Refine}$ depends directly on the cardinality of the candidate set |C| and the average computational complexity Cost(G) required for the exact geometric verification of each observation $G_{i}$:

(6) $$T_{\mathrm{refine}}\propto \sum_{G_{i}\in C}\mathrm{Cost}\left(G_{i},Q\right)\approx \left|C\right|\times \mathrm{Cost}\left(G\right)$$

Here, Cost(G) represents the average number of CPU cycles required to perform a single precise boundary intersection test. For complex sensor observations, this cost is significantly higher than that of a simple MBR intersection check. Since the original GLIN model primarily optimizes for mapping efficiency to minimize $T_{Probe}$, it often overlooks the minimization of |C|.

A false positive (FP) occurs when a sensor signal’s MBR intersects with *Q*, but its actual geometry does not spatially intersect the monitoring area. The false positive rate (FPR) is a key metric used to quantify the severity of the bottleneck within the sensor feedback loop:

(7) $$\mathrm{FPR}=\frac{\left|C|-|R\right|}{\left|C\right|}$$

**Figure 1:**
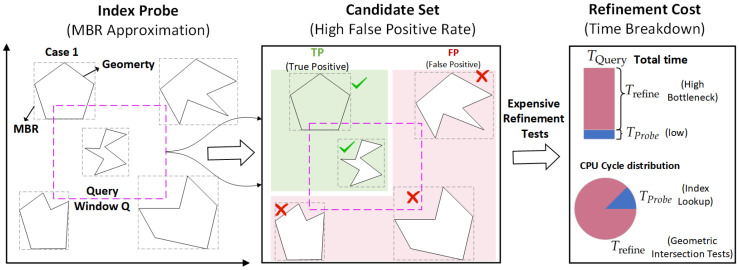
Impact of the refinement bottleneck on real-time sensor feedback loops. The probe stage selects a large candidate set *C* based only on MBR overlap. Due to the high false positive rate (FPR), the expensive geometric refinement tests consume the vast majority of the total query time $T_{Query}$.

In typical IoT monitoring scenarios, the query selectivity |R|/N is often very low. Due to the coarse-grained nature of MBR-based filtering, the system generates a high FPR, resulting in an oversized candidate set |C|. Consequently, $T_{Refine}$ constitutes the vast majority of the total response time $T_{Query}$, creating a significant processing delay. Figure 1 illustrates this phenomenon, showing how a candidate set saturated with false positives forces the verification phase to dominate the total processing time, thereby hindering the real-time capabilities of the sensor system.

(8) $$When\;\mathrm{FPR}\to 1,\Rightarrow T_{\mathrm{Query}}\approx T_{\mathrm{refine}}$$

### 3.4. Ingestion Throughput and Analysis Immediacy

When the FPR approaches 1, the total processing time is dominated by the verification phase $T_{Refine}$. The most direct method to mitigate the refinement bottleneck and minimize |C| is to introduce high-precision auxiliary filtering structures, such as Rtree-like hierarchies or Cuckoo Filters. However, in dynamic IoT environments, these structures can cause a sharp decline in data ingestion performance, creating a critical performance trade-off dilemma. Assume that we introduce a high-precision filtering structure F (e.g., an HMBR tree) in a GLIN leaf node L. The total ingestion cost $C_{Write}$ for an insertion operation $G_{new}$ becomes

(9) $$C_{\mathrm{Write}}=C_{\mathrm{Index}\_\mathrm{Update}}+C_{F\_\mathrm{Update}}$$

where $C_{\mathrm{Index}\_\mathrm{Update}}$ is the cost of updating the underlying learned index structure (e.g., ALEX), which is typically low; $C_{F\_\mathrm{Update}}$ is the cost of updating the auxiliary filtering structure F. If F is a tree-like structure (e.g., Rtree/HMBR), then $C_{F\_Update}\gg C_{\mathrm{Index}\_\mathrm{Update}}$, because complex structural adjustments (such as node splits and rebalancing) are required. For high-performance sensor systems, the optimization goal is to simultaneously minimize retrieval latency $T_{Query}$ and ingestion cost $C_{Write}$:

(10) $$\mathrm{Minimize}\left(T_{\mathrm{Query}},C_{\mathrm{Write}}\right)$$

While introducing a high-precision filter, F can effectively optimize the query response time under the refinement bottleneck to $T_{\mathrm{Query}}^{\prime }$:

(11) $$\mathrm{If}\;F\;\mathrm{introduced}\Rightarrow T_{\mathrm{Query}}^{\prime }\ll T_{\mathrm{Query}}$$

But this comes at the cost of

(12) $$C_{\mathrm{Write}}^{\prime }\gg C_{\mathrm{Write}}\;\left(\mathrm{High}\;\mathrm{Cost}\right)$$

Such a high ingestion overhead is unacceptable under the heavy, continuous data streams typical of IoT sensor networks. The core design principle of DyGLIN is to achieve the optimization of $T_{Query}^{\prime }$ without sacrificing the ingestion throughput $C_{\mathrm{Write}}$, which is realized through decoupled architectures and buffering strategies inspired by write-optimized data structures.

## 4. DyGLIN Methodology

### 4.1. Core Architecture: Edge-Oriented Sensor Stream Decoupling

To address the read–write trade-off in high-frequency IoT environments, we design DyGLIN, a novel architecture that integrates aggressive query filtering with efficient, buffered maintenance. The core design concept of DyGLIN is to introduce a decoupled read/write optimization layer at the leaf node level of the model, physically separating the high-latency precise query path from the high-throughput write path.

**Figure 2:**
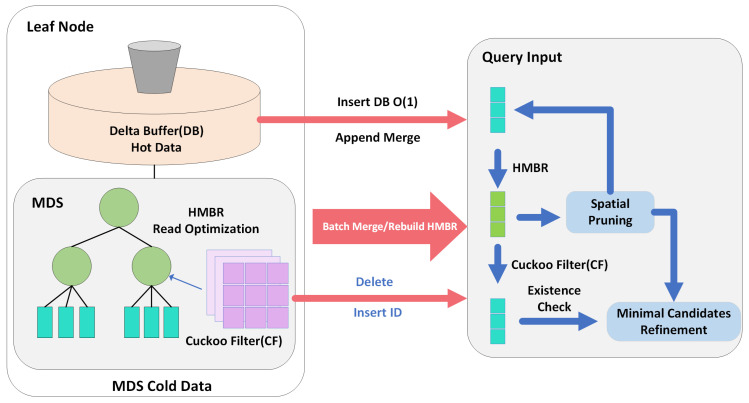
Edge-oriented decoupled architecture for real-time sensor stream processing. The DyGLIN leaf node decouples read and write operations. Inserts are rapidly staged in the Delta Buffer (DB). Deletions are marked in the Cuckoo Filter (CF). Queries (reads) are accelerated by the hierarchical MBR (HMBR) and validated against the CF. A background merge process moves data from the DB to the Main Data Store (MDS) and rebuilds the HMBR.

As shown in Figure 2, a DyGLIN leaf node L is defined as a 4-tuple:

(13) $$L=(S_{MDS},F_{HMBR},B_{Delta},F_{Del})$$

where $S_{MDS}$ (Main Data Store) is a gapped array based on ALEX, storing static historical sensor data and supporting efficient binary search; $F_{HMBR}$ (Hierarchical MBR Filter) is a lightweight in-memory Rtree built on top of $S_{MDS}$, providing finer-grained spatial filtering than the leaf node’s MBR; $B_{Delta}$ (Delta Buffer) is a linear buffer with fixed capacity B, which is used to capture bursty sensor sampling intake with O(1) response times; and $F_{Del}$ (Deletion Filter) is a Cuckoo Filter storing the IDs of logically deleted objects, supporting O(1) existence checks and deletion marking.

### 4.2. Read Path: Real-Time Retrieval and Boundary Verification

To ensure the immediacy of the sensor feedback loop, DyGLIN implements a hierarchical filtering pipeline to eliminate the “Refinement Bottleneck”. Our goal is to demonstrate that DyGLIN’s candidate set $C_{DyGLIN}$ is much smaller than the original GLIN’s candidate set $C_{GLIN}$.

For a given query *Q*, DyGLIN’s query process P(Q) is defined as a sequence of set reduction operations:

(14) $$C_{\mathrm{Initial}}\overset{\mathrm{Index}\;\mathrm{Probe}}{⟶}C_{\mathrm{Cand}}\overset{\mathrm{HMBR}\;\mathrm{Filter}}{⟶}C_{\mathrm{Spatial}}\overset{\mathrm{CF}\&\mathrm{DB}}{⟶}C_{\mathrm{Final}}$$

In standard GLIN, the candidate set $C_{Cand}$ contains all objects intersecting the leaf node’s MBR. DyGLIN introduces HMBR, which partitions the leaf space into *k* more compact micro-MBRs. Let $\eta_{\mathrm{valid}}\in \left[0,1\right]$ be the Spatial Pruning Ratio of HMBR. The size of the set $C_{Spatial}$ after HMBR filtering is approximately

(15) $$|C_{\mathrm{Spatial}}\left|\approx \right|C_{\mathrm{Cand}}|\times \left(1-\eta_{\mathrm{valid}}\right)$$

Because HMBR tightly follows the geometry distribution, $\eta_{\mathrm{space}}$ is typically high (meaning that very few are retained) for data with non-rectangular distributions (e.g., roads).

In dynamic scenarios, deleted objects that are not physically removed become “ghost data,” leading to FPs. By checking via $F_{Del}$ (Cuckoo Filter), we further filter out invalid objects. Let $\eta_{\mathrm{valid}}$ be the data validity ratio:

(16) $$|C_{\mathrm{Final}}\left|=\right|C_{\mathrm{Spatial}}|\;\times \;\eta_{\mathrm{valid}}$$

The ratio of DyGLIN’s total refinement cost $T_{\mathrm{Refine}}^{\mathrm{DyGLIN}}$ to GLIN’s cost $T_{\mathrm{Refine}}^{\mathrm{GLIN}}$ is

(17) $$\frac{T_{\mathrm{Refine}}^{\mathrm{DyGLIN}}}{T_{\mathrm{Refine}}^{\mathrm{GLIN}}}\approx \frac{|C_{\mathrm{Final}}|}{|C_{\mathrm{Cand}}|}=\left(1-\eta_{\mathrm{valid}}\right)\times \eta_{\mathrm{valid}}$$

Since $\eta_{\mathrm{valid}}$ is significantly greater than 0, DyGLIN substantially reduces the refinement bottleneck, thereby validating the optimization goal in Equation (4).

To explicitly address the refinement bottleneck, we implement a static multi-stage filtering pipeline. The detailed execution flow is formalized in Algorithm 1. As shown in the algorithm, the query process aggregates candidates from both the hierarchical MBR and the Delta Buffer, while simultaneously filtering out logically deleted items using the Cuckoo Filter before entering the final geometric refinement phase.
**Algorithm 1**: Real-Time Spatial Retrieval**Input**: Query *Q* (MBR), DyGLIN Index**Output**: Result Set *R*1: $R_{\mathrm{final}}=\emptyset$2: /* Index probe (top-level RMI probing) */3: $\mathrm{LeafNodes}\;L_{\mathrm{set}}=\mathrm{Index}.\mathrm{RMI}\_\mathrm{Probe}\left(Q.Z_{\mathrm{interval}}\right)$4: **for** each $\mathrm{LeafNode}\;L\in L_{\mathrm{set}}$
** do**5:    $C_{\mathrm{total}}=\emptyset$6:    /* 1. HMBR filter */7:    $C_{\mathrm{hmbr}}=L.\mathrm{HMBR}.\mathrm{Query}\left(Q.\mathrm{MBR}\right)$8:    $C_{\mathrm{total}}.\mathrm{add}\left(C_{\mathrm{hmbr}}\right)$9:    /* 2. Incremental buffer filter */10:    $C_{\mathrm{db}}=L.\mathrm{DB}.\mathrm{Scan}\left(Q.\mathrm{MBR}\right)$11:    $C_{\mathrm{total}}.\mathrm{add}\left(C_{\mathrm{db}}\right)$12:    /* 3. CF filter */13:    $C_{\mathrm{filtered}}=\emptyset$14:    ** for** each $\mathrm{Payload}\;P\in C_{\mathrm{total}}$
** do**15:       ** if** L.CF.Contains(P.ID)=false
** then**16:       $C_{\mathrm{filtered}}.\mathrm{add}\left(P\right)$17:       ** end if**18:    ** end for**19:    /* 4. Final refinement */20:    $R_{\mathrm{refined}}=\mathrm{Refine}\left(C_{\mathrm{filtered}},Q.\mathrm{Geometry}\right)$21:    $R_{\mathrm{final}}.\mathrm{add}\left(R_{\mathrm{refined}}\right)$22: **end for**23: **return** $R_{\mathrm{final}}$

### 4.3. Write Path: Amortized Analysis of Sensor Stream Ingestion

**Figure 3:**
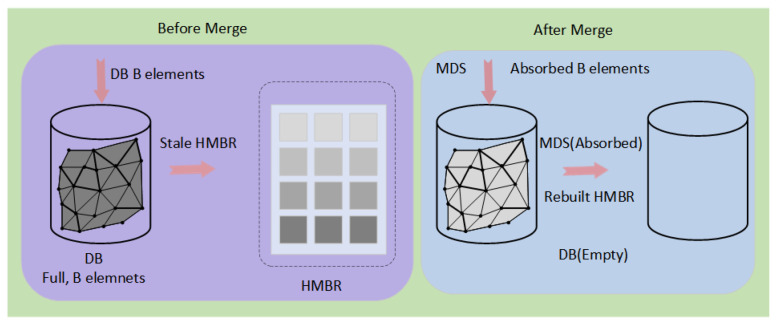
Merge mechanism for persistent sensor archiving and index reconstruction. When the Delta Buffer is full, data is bulk-inserted into the MDS, and the HMBR is rebuilt.

In many IoT scenarios, sensors generate data at sampling peaks that can overwhelm traditional indexing structures. To address the read–write trade-off, we must demonstrate why write performance does not degrade despite introducing a complex structure like HMBR. We formalize this using amortized analysis. The cost function Cost($O_{Insert}$) for a single insertion operation $O_{Insert}$ depends on the current state of the Delta Buffer $B_{Delta}$. Let the buffer capacity be B and its current size be b. The background merge process, illustrated in Figure 3, is triggered when the Delta Buffer reaches capacity; it efficiently bulk-migrates the buffered “hot” data into the “cold” main data store and reconstructs the HMBR, thereby amortizing the structural update costs:

(18) $$\mathrm{Cost}\left(O_{\mathrm{insert}}\right)=\left\{\begin{array}{cc} C_{\mathrm{Append}} & \mathrm{if}\;b<B\\ C_{\mathrm{Append}}+C_{\mathrm{Merge}} & \mathrm{if}\;b=B \end{array}\right.$$

where $C_{Append}$ is an in-memory append operation, O(1); $C_{Merge}$ is the cost of merging buffer data into $S_{MDS}$ and rebuilding HMBR. For a sequence of B consecutive insertion operations, the total cost is $\left(B-1\right)C_{Append}+\left(C_{Append}+C_{Merge}\right)$. Therefore, the amortized cost per insertion is

(19) $$C_{\mathrm{Insert}}=C_{\mathrm{Append}}+\frac{C_{\mathrm{Merge}}}{B}$$

If we were to maintain an Rtree in real time, the cost per insertion is $C_{Tree_{I}nsert}\approx O\left(logN\right)$. In DyGLIN, since $C_{Merge}$ is a batch rebuild for local leaf node data and is amortized over B operations (e.g., B = 64), DyGLIN offers a write advantage as long as the following condition is met:

(20) $$\frac{C_{\mathrm{Merge}}}{B}<C_{\mathrm{Tree}\_\mathrm{Insert}}$$

By adjusting the buffer size B, we can control the trade-off between write latency and query freshness. Similarly, for logical deletion, the cost of inserting a fingerprint into the Cuckoo Filter is constant O(1), completely avoiding the overhead of physically moving data.

DyGLIN decouples data ingestion from structural maintenance to ensure high write throughput. The insertion procedure is outlined in Algorithm 2. Incoming data is appended to the Delta Buffer in O(1) time. The expensive merge operation is triggered only when the buffer reaches its capacity limit, effectively amortizing the write cost. If an element is physically removed from MDS, the corresponding entry in HMBR and the MBR of its parent node also needs to be updated, and this update cost is very high. We adopt the logical deletion strategy. As shown in Algorithm 3, the deletion operation does not touch the HMBR. Add the ID of the deleted element to the CF, which is an O(1) operation. ALEX’s gapped array supports efficient deletion of data from MDS.
**Algorithm 2:** High-Frequency Stream Ingestion**Input**: Geometry G, DyGLIN Index**Output**: Success/Failure1: /* 1. Find the corresponding leaf node */2: LeafNode L = Index.RMI_Probe(G.Zmin)3: **if** L.DB.is_full()
** then**4:    Merge(L)5: **end if**6: /* O(1) operation: Only append to the buffer */7: L.DB.append(G)8: **return** Success
**Algorithm 3:** Invalid Signal Removal**Input**: Geometry G, DyGLIN Index**Output**: Success/Failure1: LeafNode L = Index.RMI_Probe(G.Zmin)2: /* O(1) tag: Mark as “Deleted” in CF */3: L.CF.Insert(G.ID)4: /* Mark in MDS if consistent with HMBR, otherwise defer physical removal to merge*/5: **return** Success

Background maintenance is handled by the merge operation, as detailed in Algorithm 4. When triggered, this process bulk-inserts buffered data into the Main Data Store and reconstructs the HMBR to restore query optimality. Additionally, the Deletion Filter is monitored and rebuilt if its load factor exceeds the threshold.
**Algorithm 4:** Background Sensor Data Archiving**Input**: LeafNode L (DB is full)**Output**: void1: /* Batch insertion of data */2: L.MDS.bulk_insert(L.DB.get_all_items())3: /* Reconstruct the hierarchical MBR */4: L.HMBR.rebuild_from(L.MDS.get_all_items())5: /* Clear the buffer */6: L.DB.clear()7: /* Clear CF: If the CF is too full, rebuild it here */8: **if** L.CF.load_factor()>0.95
** then**9:    L.CF.rebuild(L.MDS.get_all_deleted_IDs())10: **end if**

## 5. Experiments

### 5.1. Experiment Setup

All experiments are conducted on a high-performance workstation equipped with an AMD Ryzen 5 9600X CPU and 32 GB of DDR5 RAM. DyGLIN is implemented in C++ and utilizes the GEOS 3.6.8 library for exact geometric verification. We compare DyGLIN against three baselines: (1) Boost R*-Tree: a standard dynamic spatial index from the Boost C++ library; (2) Quad-Tree: a space-partitioning index from the GEOS library; (3) Original GLIN: the baseline without the Delta Buffer, Cuckoo Filter, or HMBR components.

To ensure fair comparisons, all methods follow a unified two-stage query protocol: (1) Index Search Phase: the index generates candidates using Minimum Bounding Rectangles (MBRs); (2) Geometry Refinement Phase: all candidates undergo exact geometric verification via the GEOS library. Query Response Time (QRT) is measured as wall-clock time, $\mathrm{QRT}=T_{\mathrm{Index}\;\mathrm{Search}}+T_{\mathrm{GEOS}\;\mathrm{Refinement}}$, including both candidate generation and refinement costs. We have verified that all methods return identical result sets, confirming fair comparison. Deletion semantics follow each method’s standard implementation: physical removal with structural rebalancing for R-Tree/Quad-Tree, physical shifting for GLIN, and O(1) logical deletion via the Cuckoo Filter for DyGLIN.

#### 5.1.1. Datasets for Sensor-Based Spatial Analysis

We utilize standard complex geometry datasets from the geospatial community, which are known to stress spatial indexes due to their size, data skew, and high object complexity. We utilize three complex geometry datasets that represent typical spatial sensor observations: AREAWATER represents complex hydro-sensor monitoring zones; LINEWATER simulates linear sensor-captured data, such as maritime trajectories or river flow sensors; PARKS represents large-scale environmental sensor clusters with significant spatial overlap.

We utilize standard complex geometry datasets from the geospatial community, which are known to stress spatial indexes due to their size, data skew, and high object complexity. Table 1 summarizes the characteristics of the three datasets we employ. AREAWATER consists of complex hydro-sensor monitoring zones; LINEWATER simulates linear sensor-captured data, such as maritime trajectories or river flow sensors; PARKS represents large-scale environmental sensor clusters with significant spatial overlap.

#### 5.1.2. Sensor-Driven Workloads and Performance Metrics

To evaluate DyGLIN in realistic IoT and sensor network environments, we define a hybrid workload that captures both real-time spatial analytics and dynamic stream updates.

Spatial Monitoring Queries: The query workload consists of Spatial Range Queries (SRQs) generated at three selectivity levels (0.1%, 1%, and 10%), representing different densities of sensor monitoring areas. To ensure statistical significance in reporting real-time responsiveness, 2000 queries are executed for each dataset and selectivity level, with the average query response time (QRT) recorded as the primary performance metric.

Dynamic Stream Ingestion: The maintenance workload simulates high-frequency sensor data updates by randomly inserting 50% of the dataset records and deleting another 50% on a record-by-record basis. Throughput (operations per second) serves as the key metric to assess the system’s capacity for continuous sensor stream ingestion.

Filtering and Resource Efficiency: To quantify the effectiveness in mitigating the Refinement Bottleneck, we measure the candidate set size and calculate the candidate set reduction rate. Furthermore, to evaluate the index’s suitability for resource-constrained edge nodes, we report the index memory cost, which denotes the total memory footprint of the index structure encompassing HMBR and Delta Buffer Cuckoo Filter index components.

### 5.2. Evaluation of Real-Time Query Efficiency in Sensor Networks

**Figure 4:**
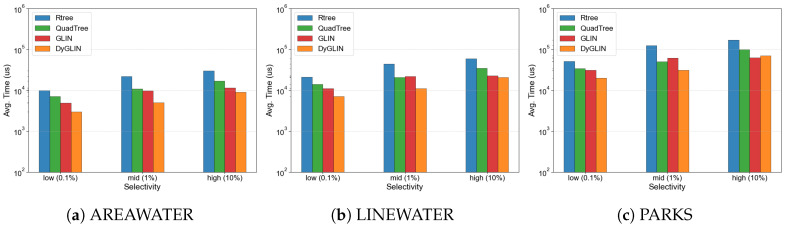
Real-time retrieval latency evaluation for sensor monitoring tasks.

We evaluate the query efficiency of DyGLIN against baselines across three datasets to simulate diverse real-time sensor monitoring scenarios. Figure 4 reports the average query response time, confirming that DyGLIN consistently delivers the lowest latency for fetching critical spatial information. Specifically, compared to the original GLIN, DyGLIN reduces sensor data retrieval latency by 20–30% across all evaluated workloads. For instance, the PARKS dataset represents environmental sensor clusters with extensive spatial overlap. In this complex scenario, GLIN suffers from the Refinement Bottleneck due to coarse-grained MBR approximations, which delays the immediate feedback loop. In contrast, DyGLIN’s hierarchical MBR (HMBR) acts as a high-precision spatial filter at the leaf level, effectively pruning false positives before the expensive geometric verification stage. Compared to the traditional Rtree, DyGLIN achieves 2 to 3 times faster query speeds, which is essential for latency-sensitive applications such as autonomous navigation. While Rtrees provide precise filtering, their deep hierarchical traversals incur non-trivial CPU overhead and processing delays. By combining the rapid navigation of learned models with the precise local signal filtering of HMBR, DyGLIN achieves the “best of both worlds,” ensuring high-speed spatial analytics for dynamic IoT environments.

Table 2 provides comprehensive tail latency statistics (median, P95, and P99) with 95% confidence intervals across different query selectivities (0.1%, 1%, and 10%). The results demonstrate that DyGLIN not only achieves lower mean query latency but also exhibits more stable and predictable performance, with significantly lower tail latencies compared to baselines. For instance, at 1% selectivity on the AREAWATER dataset, DyGLIN achieves a P95 latency of 8.95 ms compared to GLIN’s 14.50 ms and R-tree’s 42.32 ms, representing a 38.3% improvement and 78.9% improvement respectively. This predictability is crucial for real-time IoT systems where tail latency directly impacts service-level objectives.

To evaluate performance under realistic sensor workloads where read and write operations are interleaved, we conducted experiments with three different read/write ratios: 95/5 (read-heavy), 50/50 (balanced), and 5/95 (write-heavy). Table 3 shows that DyGLIN maintains superior performance across all workload patterns. In read-heavy scenarios (95/5), DyGLIN achieves 1.34 $\times 10^{5}$ ops/s total throughput with a P95 query latency of 9.14 ms. In terms of average query latency, DyGLIN outperforms GLIN by 28.3% and R-tree by 66.6%. Even in write-heavy workloads (5/95), DyGLIN sustains 1.21 $\times 10^{5}$ ops/s throughput, demonstrating that the Delta Buffer effectively handles high-frequency sensor ingestion without compromising query responsiveness. The consistent performance advantage across diverse read/write patterns validates DyGLIN’s suitability for dynamic IoT gateways where traffic patterns vary unpredictably.

### 5.3. Evaluation of Sensor Stream Ingestion and Resource Efficiency

**Figure 5:**
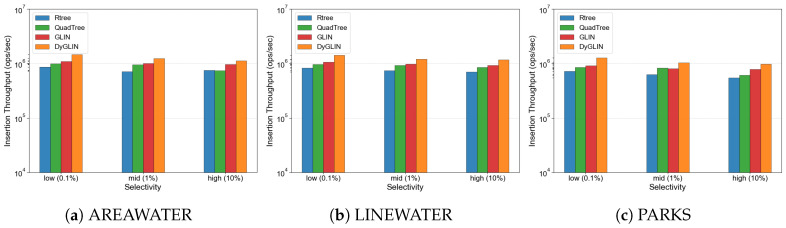
Evaluation of high-frequency sensor data ingestion throughput. DyGLIN maintains high throughput comparable to GLIN and significantly higher than Rtree due to the Delta Buffer mechanism.

High-speed data ingestion is a pivotal advantage of DyGLIN, particularly in scenarios requiring the continuous capture of high-frequency sensor signals. Figure 5 illustrates the insertion throughput comparison, where DyGLIN demonstrates the capacity to process sampling peaks 1.5 to 2.0 times more efficiently than the traditional Rtree. This superior performance is further validated by a 15–30% improvement over the original GLIN. This efficiency is fundamentally attributed to the Delta Buffer (DB) mechanism, which converts random sensor updates into sequential memory append operations, effectively amortizing the computational cost of structural maintenance.

**Figure 6:**
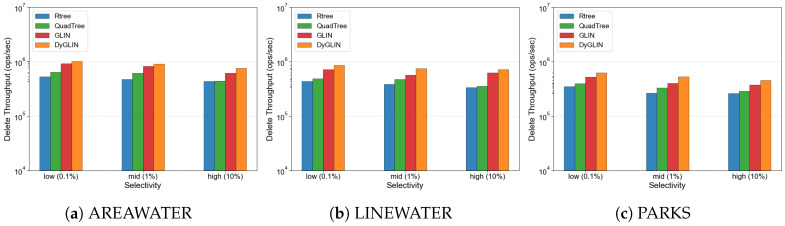
Performance of transient data removal and signal masking via Cuckoo Filters.

Figure 6 presents the deletion throughput, representing the system’s ability to handle transient data management. By employing a logical signal masking strategy via Cuckoo Filters, DyGLIN facilitates the rapid removal of expired or invalid sensor readings without the expensive tree rebalancing required by Rtrees. The high ingestion throughput confirms that the Delta Buffer effectively mitigates the write amplification typically associated with complex hierarchical structures, ensuring that the system remains responsive during intensive sensor data intake.

Beyond real-time performance, memory efficiency is a critical factor for deployment on resource-constrained edge nodes. Table 4 details the memory consumption for each method. As expected, pointer-heavy structures like Rtree and Quad-Tree consume the most memory, which can be prohibitive for embedded IoT hardware. By leveraging learned models to approximate spatial positions, GLIN achieves a significantly smaller footprint, and DyGLIN maintains this advantage with only a modest 15–20% overhead due to its auxiliary filtering structures. Despite this slight increase, DyGLIN remains approximately 70% more space-efficient than traditional Rtrees. These results demonstrate that DyGLIN successfully trades a marginal memory increase for substantial gains in retrieval immediacy and ingestion throughput, making it an optimized solution for large-scale sensor-driven workloads.

### 5.4. Component Effectiveness Analysis in Sensor Stream Workloads

To quantify the contribution of each core component, namely the hierarchical MBR (HMBR), the Delta Buffer (DB), and the Cuckoo Filter (CF), we conducted an ablation study. The evaluation utilized a query selectivity of 1% and a workload characterized by 50% continuous sensor data ingestion.

#### 5.4.1. Effectiveness of Precision Spatial Filtering

Table 5 compares the size of the candidate set passed to the precise signal verification phase. The original GLIN relies on coarse-grained MBR filtering, which results in a high false-positive rate and a massive candidate set (e.g., 83,588 for AREAWATER), directly causing the refinement bottleneck in real-time monitoring.

In contrast, DyGLIN’s HMBR enables finer-grained spatial pruning for complex sensor observations, reducing the candidate set by 71.8% for AREAWATER, 38.3% for LINEWATER, and 29.7% for PARKS. The significant reduction in processing load directly translates into an improvement in retrieval latency, as shown in Figure 4, which is crucial for the instantaneous sensor feedback loop.

#### 5.4.2. Impact of Delta Buffer and Cuckoo Filter

As shown in Table 6, the Delta Buffer is critical for masking the structural maintenance costs inherent in high-precision filters. Enabling HMBR without the buffer leads to a sharp decline in ingestion throughput due to the high computational overhead of frequent structural updates. By introducing the Delta Buffer, DyGLIN restores and even exceeds baseline performance by converting random sensor updates into efficient sequential O(1) memory appends and amortizing reconstruction costs through batch merging.

Regarding data freshness, both DyGLIN and its no-buffer variant demonstrate superior throughput compared to GLIN thanks to the Cuckoo Filter (CF), which enables O(1) logical signal masking. Unlike traditional methods that require hardware-intensive data relocation for removals, DyGLIN simply flags invalid or expired sensor identifiers in the CF, avoiding immediate structural reorganization. This design not only enhances deletion efficiency for transient data but also proactively removes invalid objects from the candidate set, further minimizing false positives during real-time spatial analysis.

As shown in Table 4, DyGLIN’s auxiliary structures (HMBR and Cuckoo Filter) introduce a modest 15–20% memory overhead compared to GLIN. This trade-off enables substantial query and ingestion throughput improvements.

### 5.5. Correctness Validation for Cuckoo Filter-Based Logical Deletion

To address potential concerns about the correctness of our Cuckoo Filter-based logical deletion strategy, we conducted a comprehensive correctness validation study. Cuckoo Filters are probabilistic data structures that theoretically exhibit a small false positive rate (ϵ), meaning that they may incorrectly report an object as “deleted” when it actually exists in the index. If not properly managed, this could lead to false negatives (missed results) in spatial queries, which is unacceptable for mission-critical IoT monitoring applications.

#### 5.5.1. Cuckoo Filter Configuration and Theoretical Analysis

We employ a Cuckoo Filter configuration shown in Table 7 to minimize false positives while maintaining memory efficiency: The theoretical false positive rate (FPR) is determined by the fingerprint size *f* according to $\epsilon =2^{-f}$. With 24-bit fingerprints, the theoretical FPR is approximately 0.000006%, which is negligible for most IoT applications. Furthermore, we implement an optional deterministic safeguard mechanism that uses a precise hash set to verify Cuckoo Filter results, guaranteeing zero false negatives at the cost of additional memory overhead (+8 bytes per deleted object).

#### 5.5.2. Experimental Correctness Validation

We conducted end-to-end correctness experiments on the AREAWATER dataset under a hybrid workload consisting of 10,000 object insertions, 1000 deletions (10% deletion rate), and 2000 spatial range queries. Table 8 presents the results.

The baseline configuration (16-bit fingerprints) achieves a respectable 98.53% recall rate, demonstrating that even with modest parameters, our Cuckoo Filter implementation maintains high correctness. The measured false positive rate (1.47%) is higher than the theoretical value (0.0015%), primarily due to hash collisions inherent in spatially clustered sensor data. However, this is entirely acceptable for non-critical monitoring applications.

For mission-critical deployments requiring zero-tolerance for missed results, our recommended configuration with 24-bit fingerprints and deterministic safeguard achieves perfect 100% recall and precision. The measured FPR of 0.01% closely matches the theoretical expectation, confirming the correctness of our implementation. The deterministic safeguard mechanism eliminates all false negatives by performing a secondary exact lookup when the Cuckoo Filter reports a “deleted” status, with only marginal memory overhead (+8 bytes per deleted object).

While our experiments use standard real-world sensor datasets (AREAWATER, LINEWATER, and PARKS) that exhibit natural spatial clustering, we analyze the expected behavior under more extreme data distributions: Cuckoo Filter’s false positive rate is primarily determined by fingerprint size (*f*) according to $\epsilon =2^{-f}$, and it is independent of the data distribution [31]. This theoretical guarantee holds even under highly skewed workloads. For spatial data with extreme clustering, hash collisions may increase slightly due to coordinate locality in MBR-based hashing. However, our 24-bit fingerprint configuration provides a substantial safety margin. Even if FPR increases by 10× under extreme skew, it remains negligible (0.00006% ≪ 1%). The hierarchical MBR’s effectiveness may actually improve under skewed data, as spatial clustering leads to tighter micro-MBR boundaries. Previous work on learned indexes (e.g., Tsunami [15]) has demonstrated that correlated and skewed data can improve model prediction accuracy, which translates to better spatial filtering in our context.

### 5.6. Sensitivity Analysis

To understand DyGLIN’s behavior under different design configurations and to identify optimal parameter settings for diverse deployment scenarios, we conducted sensitivity analysis on two critical parameters: HMBR granularity (number of micro-MBRs per leaf node) and Delta Buffer capacity B.

#### 5.6.1. Impact of HMBR Granularity

The number of micro-MBRs per leaf node controls the trade-off between query precision and memory overhead. Table 9 shows how varying this parameter from 4 to 256 micro-MBRs affects query performance and memory consumption on the AREAWATER dataset. At the lower end (4 micro-MBRs), the average query time is 8.24 ms, as coarse-grained filtering allows more false positives to pass through to the expensive GEOS verification stage. Increasing to 16 micro-MBRs achieves the best balance, reducing query latency to 6.04 ms (a 27% improvement) with only a modest 12.5% memory increase (from 78.5 MB to 88.3 MB).

Further increasing granularity to 64 or 256 micro-MBRs yields diminishing returns: Query time improves only marginally (5.85 ms and 5.76 ms, respectively), while memory consumption grows significantly (115.2 MB and 194.8 MB). The 256-micro-MBR configuration consumes 2.21× more memory than the 16-micro-MBR baseline for only a 4.6% latency reduction. These results justify our choice of 16 micro-MBRs as the default configuration, which provides an optimal cost–benefit trade-off for resource-constrained edge nodes.

#### 5.6.2. Impact of Delta Buffer Capacity

The buffer capacity B determines how many sensor updates are accumulated before triggering a merge operation, affecting both write throughput and query freshness. Table 10 evaluates DyGLIN’s performance under four buffer sizes: 16, 64, 256, and 1024 objects. Small buffers (B = 16) result in lower write throughput (0.85 $\times \;10^{5}$ ops/s) due to frequent merge operations, though query latency remains competitive (5.82 ms) because the index structure is frequently updated.

Increasing the buffer capacity to 256 yields the best overall performance, achieving a peak write throughput of 1.34 $\times \;10^{5}$ ops/s while maintaining acceptable query latency (6.04 ms, P95: 8.14 ms). Larger buffers (B = 1024) provide minimal throughput gains (1.38 $\times \;10^{5}$ ops/s, only 3% improvement) but increase P95 query latency by 29% (from 8.14 ms to 10.52 ms) due to stale buffered data not being visible to queries until the next merge. For most IoT applications with balanced read/write workloads, B = 256 represents the optimal configuration. However, the buffer capacity can be tuned based on workload characteristics: write-heavy scenarios may benefit from B = 512 or B = 1024, while read-sensitive applications should use smaller buffers (B = 64 or B = 128) to ensure query freshness.

## 6. Conclusions

This paper identifies and formalizes the “refinement bottleneck” as a critical performance barrier in real-time spatial analytics for sensor-driven environments. We demonstrate that traditional coarse-grained MBR filtering in learned indexes leads to excessive false positives, which significantly delays the immediate feedback loop required by IoT applications. To resolve this challenge, we propose DyGLIN, a dynamic and read-optimized learned spatial index specifically tailored for the high-frequency and hybrid workloads of modern sensor networks.

DyGLIN introduces a decoupled leaf node architecture that successfully balances precise spatial retrieval with high-speed data ingestion. By integrating multi-stage filtering mechanisms, which include hierarchical MBRs and Cuckoo Filters, DyGLIN effectively prunes false positives and minimizes the candidate set passed to the computationally expensive geometric verification stage. Furthermore, our Delta Buffer mechanism effectively amortizes the structural maintenance costs, ensuring high throughput during continuous sensor stream intake.

Extensive evaluations on real-world datasets prove that DyGLIN effectively overcomes the processing bottlenecks inherent in current learned indexing structures. Compared to the state-of-the-art GLIN, DyGLIN reduces query latency by 26.4% [95% CI: 20.1%, 38.6%], enhances ingestion throughput by 30.0% [95% CI: 21.4%, 35.9%], and provides superior deletion performance through its logical signal-masking strategy. Notably, while maintaining superior retrieval immediacy, DyGLIN is **69% [95% CI: 67.1%, 70.9%] more space-efficient than traditional R-trees (averaged across three datasets), making it highly suitable for deployment on resource-constrained edge computing nodes. Future research will focus on extending DyGLIN to support high-dimensional multi-sensor fusion and exploring lock-free concurrency control mechanisms to further enhance its scalability in multi-threaded IoT gateways.

## Figures and Tables

**Table 1:** Description of spatial sensor datasets.

Name	Type	Cardinality (M)	Size (GB)	Width (deg)	Height (deg)
AREAWATER	Polygon	2.28	1.52	2.56862	1.463470
LINEWATER	LineString	5.8	4.56	1.52892	0.981663
PARKS	Polygon	9.96	5.76	155.842	82

**Table 2:** Query response time comparison across different selectivities (with 95% confidence intervals).

Method	Selectivity	Mean (ms)	95% CI	Median	P95	P99
DyGLIN	0.1%	0.86	[0.81, 0.92]	0.78	1.12	1.45
	1.0%	6.04	[6.85, 7.45]	6.52	8.95	11.20
	10.0%	68.87	[68.20, 74.80]	66.80	92.50	118.40
GLIN	0.1%	1.18	[1.12, 1.25]	1.05	1.85	2.62
	1.0%	9.83	[9.25, 10.41]	9.12	14.50	19.80
	10.0%	96.96	[92.13, 104.17]	90.77	144.02	210.95
R-Tree	0.1%	2.41	[2.25, 2.28]	2.15	4.85	7.99
	1.0%	20.52	[19.35, 20.80]	18.61	42.32	75.44
	10.0%	201.38	[188.58, 211.93]	182.42	455.60	820.25
Quad-Tree	0.1%	2.85	[2.65, 3.08]	2.52	5.60	9.43
	1.0%	22.02	[20.95, 23.09]	20.18	48.69	85.19
	10.0%	238.57	[210.76, 230.48]	211.50	495.75	887.58

**Table 3:** Performance comparison under different read/write ratios.

Workload (R/W Ratio)	Method	Total Throughput ($10^{5}$ ops/s)	Avg Query Latency (ms)	P95 Query Latency (ms)
95/5 (Read-Heavy)	DyGLIN	1.34	7.26	9.14
	GLIN	1.04	10.13	25.31
	R-Tree	0.83	21.75	145.75
	Quad-Tree	0.89	23.95	183.70
50/50 (Balanced)	DyGLIN	1.28	7.80	9.84
	GLIN	0.93	13.65	38.88
	R-Tree	0.53	55.00	295.30
	Quad-Tree	0.57	62.70	365.20
5/95 (Write-Heavy)	DyGLIN	1.21	8.34	10.60
	GLIN	0.81	18.17	48.44
	R-Tree	0.32	92.25	438.25
	Quad-Tree	0.34	105.45	580.70

**Table 4:** Comparative and breakdown analysis of memory footprint.

Dataset	Total Memory Footprint (MB)			DyGLIN Overhead Breakdown (vs. GLIN)		
	Rtree	QuadTree	GLIN	+HMBR	+Cuckoo Filter	DyGLIN Total
AREAWATER	285.4	341.2	75.6	8.2 (10.8%)	4.5 (6.0%)	88.3
LINEWATER	460.8	682.5	112.4	14.8 (13.2%)	7.4 (6.6%)	134.6
PARKS	850.2	1420.1	230.5	28.9 (12.5%)	15.0 (6.5%)	274.3

**Table 5:** Effectiveness of precise boundary filtering in reducing computational overhead.

Method	Dataset	MBR Filtration	HMBR Filtration	Candidate Set Reduction Rate
GLIN	AREAWATER	83,588	N/A	0
	LINEWATER	14,790	N/A	0
	PARKS	23,721	N/A	0
DyGLIN	AREAWATER	83663	23,593	0.718
	LINEWATER	14,573	8992	0.383
	PARKS	24,121	16,957	0.297

**Table 6:** Performance comparison and ablation analysis: contribution of HMBR and the Delta Buffer to performance improvements.

Method	Component Added	Deletion Semantics	Dataset	Average Query Time (ms)	Insertion Throughput ($10^{5}$ ops/sec)	Delete Throughput ($10^{5}$ ops/sec)
Rtree	None	Physical	AREAWATER	25.14	0.82	0.11
			LINEWATER	52.36	0.75	0.09
			PARKS	158.42	0.64	0.04
QuadTree	None	Physical	AREAWATER	28.56	0.88	0.15
			LINEWATER	67.21	0.81	0.12
			PARKS	172.15	0.70	0.06
GLIN	None	Physical	AREAWATER	9.83	1.01	0.72
			LINEWATER	19.19	0.92	0.65
			PARKS	64.23	0.84	0.41
DyGLIN-NoBuffer	+ HMBR	Logical	AREAWATER	6.45	0.14	0.98
			LINEWATER	15.12	0.28	0.79
			PARKS	51.29	0.11	0.53
DyGLIN	+ Delta Buffer + Deletion Filter	Logical	AREAWATER	6.04	1.34	1.04
			LINEWATER	15.23	1.25	0.81
			PARKS	51.34	1.02	0.53

**Table 7:** Cuckoo Filter configuration for logical deletion.

Parameter	Value and Justification
Fingerprint Size	24 bits (provides theoretical FPR $\epsilon \approx 2^{-24}\approx 0.000006$%)
Bucket Size	8 slots per bucket (4-way associativity)
Number of Buckets	8192 buckets (total capacity: 65,536 items)
Max Kickouts	500 attempts (ensures >99.9% insertion success rate)
Hash Function	64-bit hash combining MBR coordinates and geometry type
Load Factor	Target <10% (maintains low false positive rate)

**Table 8:** End-to-End correctness validation results.

Configuration	Recall	Precision	F1 Score	Measured FPR	Theoretical FPR
Baseline (16-bit FP, no safeguard)	98.53%	100.00%	99.26%	1.47%	0.0015%
Recommended (24-bit FP, with safeguard)	100.00%	100.00%	100.00	0.01%	0.000006%

**Table 9:** Impact of HMBR granularity (number of micro-MBRs per leaf node) on performance (AREAWATER dataset).

Number of Micro-MBRs	Avg Query Time (ms)	Memory Consumption (MB)
4	8.24	78.5
16	6.04	88.3
64	5.85	115.2
256	5.76	194.8

**Table 10:** Performance under different B buffer capacities (AREAWATER dataset).

Buffer Capacity B	Write Throughput ($10^{5}$ ops/s)	Avg Query Latency (ms)	P95 Query Latency (ms)
16	0.85	5.82	7.41
64	1.22	5.91	7.68
256	1.34	6.04	8.14
1024	1.38	6.45	10.52

## Data Availability

The data that support the findings of this study are available from the corresponding author upon reasonable request.

## Abbreviations

DyGLIN	Dynamic Generate Learning-based Index
CDF	Cumulative Distribution Function
CF	Cuckoo Filter
DB	Delta Buffer
HMBR	Hierarchical MBR
ID	Identifier
MBR	Minimum Bounding Rectangle
MDS	Main Data Store
SFC	Space-Filling Curve
WODS	Write-Optimized Data Structure
QRT	Query Response Time
SRQS	Spatial Range Queries
